# Cellular diversity and gene expression profiles in the male and female brain of *Aedes aegypti*

**DOI:** 10.1186/s12864-022-08327-9

**Published:** 2022-02-10

**Authors:** Yingjun Cui, Susanta K. Behura, Alexander W. E. Franz

**Affiliations:** 1 Department of Veterinary Pathobiology, University of Missouri, Columbia, MO 65211 USA; 2 Division of Animal Sciences, University of Missouri, Columbia, MO 65211 USA; 3 Institute of Data Science and Informatics, University of Missouri, Columbia, MO 65211 USA

**Keywords:** *Aedes aegypti*, Mosquito, Male, Female, Brain, Single-nucleus RNA sequencing, scRNA-Seq, Sexual dimorphism, Sex determination, Nix, Doublesex

## Abstract

**Background:**

*Aedes aegypti* is a medically-important mosquito vector that transmits arboviruses including yellow fever, dengue, chikungunya, and Zika viruses to humans. The mosquito exhibits typical sexually dimorphic behaviors such as courtship, mating, host seeking, bloodfeeding, and oviposition. All these behaviors are mainly regulated by the brain; however, little is known about the function and neuron composition of the mosquito brain. In this study, we generated an initial atlas of the adult male and female brain of *Ae. aegypti* using 10xGenomics based single-nucleus RNA sequencing.

**Results:**

We identified 35 brain cell clusters in male and female brains, and 15 of those clusters were assigned to known cell types. Identified cell types include glia (astrocytes), Kenyon cells, (ventral) projection neurons, monoaminergic neurons, medulla neurons, and proximal medulla neurons. In addition, the cell type compositions of male and female brains were compared to each other showing that they were quantitatively distinct, as 17 out of 35 cell clusters varied significantly in their cell type proportions. Overall, the transcriptomes from each cell cluster looked very similar between the male and female brain as only up to 25 genes were differentially expressed in these clusters. The sex determination factor *Nix* was highly expressed in neurons and glia of the male brain, whereas *doublesex* (*dsx*) was expressed in all neuron and glia cell clusters of the male and female brain.

**Conclusions:**

An initial cell atlas of the brain of the mosquito *Ae. aegypti* has been generated showing that the cellular compositions of the male and female brains of this hematophagous insect differ significantly from each other. Although some of the rare brain cell types have not been detected in our single biological replicate, this study provides an important basis for the further development of a complete brain cell atlas as well as a better understanding of the neurobiology of the brains of male and female mosquitoes and their sexually dimorphic behaviors.

**Supplementary Information:**

The online version contains supplementary material available at 10.1186/s12864-022-08327-9.

## Background

Mosquitoes are hematophagous insects, exhibiting highly complex adaptive behaviors related to feeding, development, reproduction, and environmental interactions [1, 2]. They also display a strong sexual dimorphism represented by sex-specific physical, physiological, and behavioral traits in relation to reproduction. For example, anautogenous female mosquitoes develop and oviposit batches of eggs only when they have acquired a sufficient bloodmeal from a vertebrate host, whereas males are incapable of feeding on blood and therefore survive on carbohydrates obtained from flower nectar and honeydew [3, 4]. As a consequence of their sexually dimorphic bloodfeeding behavior, female mosquitoes, when feeding on an infected vertebrate, can acquire and eventually transmit medically-important pathogens such as *Plasmodium*, or arboviruses including yellow fever, dengue, Zika, and chikungunya viruses [5, 6]. Sexually dimorphic behaviors are the outcome of differential gene expression patterns that are initiated during insect development [7]. Investigating gene expression patterns and cellular compositions of the brain of male and female mosquitoes helps to better understand the basis of sexual dimorphic behavioral differences. An initial investigation of the neuro-transcriptome of *Ae. aegypti* via RNA-Seq has been previously performed [8]. The same research group also generated a three-dimensional reconstruction of the brain of *Ae. aegypti* to better understand the neural basis of mosquito behavior.

The brain of *Ae. aegypti* consists of approximately 248,000 cells, with approximately 220,000 of those being neurons and half of those originating from the optic lobes [9]. So far, no statistical difference (*p* = 0.119) in the total number of brain cells between males (242,670 cells +/− 4300) and females (255,240 cells +/− 6570) has been observed. Similarly, the brain of *Drosophila* consists of ~ 200,000 neurons, with about half of those originating from the optical lobe [9]. These are remarkable similarities considering that the ancestral lines of mosquitoes and fruit fly are thought to have separated ∼250 million years ago. However, being hematophagous, mosquitoes exhibit behaviors, which are clearly different from those of *Drosophila*. Several different functional regions have been identified and mapped to the female brain of *Ae. aegypti* including optic lobes, antennal lobes, ellipsoid body, fan shaped body, suboesophageal ganglion, lamina, lobula, lobula plate, medulla, and mushroom bodies, resembling those identified in *Drosophila* (https://www.mosquitobrains.org/). Lamina, medulla, lobula, and lobula plate are the four subdivisions/neuropils forming the optic lobe [10]. In *Drosophila*, the main function of the lamina is the processing of visual information including contrast enhancement during local motion [11]. The medulla responds to motion and possibly color information processing, while the lobula is processing information from large areas of the field of vision [10]. There are three major neuron types in the optic lobe, photoreceptor neurons, intrinsic neurons, and projection neurons (PN) [12, 13]. The intrinsic neuron activity is restricted to the optic lobe, while PN connect the optic lobe with the central brain. The lamina possesses intrinsic neurons, photoreceptor neurons, and lamina wide-field neurons. The local neurons of the medulla include intrinsic neurons, distal medulla (Dm) and proximal medulla (Pm) neurons. Additional medulla neurons include the transmedullary neurons (TmY1-12) projecting to the lobula/lobula plate, and the tangential neurons (Mt1-11). The lobula contains visual projection neurons termed lobular columnar cells that help flies to respond appropriately to visual stimuli [14]. Only (winged) insects possess a lobula plate containing a system of directionally selective motion sensitive interneurons [15]. As the main motion coordination center, the lobula plate is likely involved in flight coordination [16]. The mushroom bodies represent the brain’s center for cognitive processing (decision making) [17]. It is also responsible for olfactory learning (memory) in regard to odor information received by the antennal lobes via GABAergic (ventral) projection neurons (vPN) [18, 19]. Kenyon cells (KC) are the intrinsic neurons of the mushroom bodies and process the received odor information.

Recently, Croset et al. [20], reported an initial analysis of thousands of individual cells from the *Drosophila* midbrain using Drop-seq. As a result, 30 cell clusters were identified and annotated. Single-cell molecular signatures were assigned to each relevant cell type and brain region and the main fast-acting transmitters used by each cell cluster defined. Large-scale single-cell RNA sequencing was used to characterize the extensive cellular diversity in the *Drosophila* optic lobes [21]. In the study, 52 cell clusters were assigned and validated. Using RNA sequencing of FACS-sorted single-cell types, cell cluster-specific genes were identified. A single-cell transcriptome atlas of the entire adult *Drosophila* brain sampled across its lifespan was then presented by Davie et al. [22]. The authors identified 87 initial cell clusters that were further sub-clustered and validated via targeted cell sorting. Furthermore, gene expression patterns of Kenyon cells (KCs), olfactory projection neurons, ellipsoid body ring neurons, monoaminergic neurons, astrocytes, and other glia were profiled.

Here, we established a comparative profiling of gene expression patterns in the brain of male and female *Ae. aegypti* mosquitoes at single-cell (nucleus) resolution. We generated an initial cell atlas of the *Ae. aegypti* brain using 10xGenomics based snRNA-Seq and revealed a major distinction between the brains of the two sexes in regard to their cell type compositions. Gene expression profiles were compared between the cell clusters of the male and female brains and differentially expressed genes were identified.

## Results

### Using single-nucleus transcriptomics (snRNA-Seq) to identify individual cell types in the brains of male and female *Ae. aegypti*

**Fig. 1:**
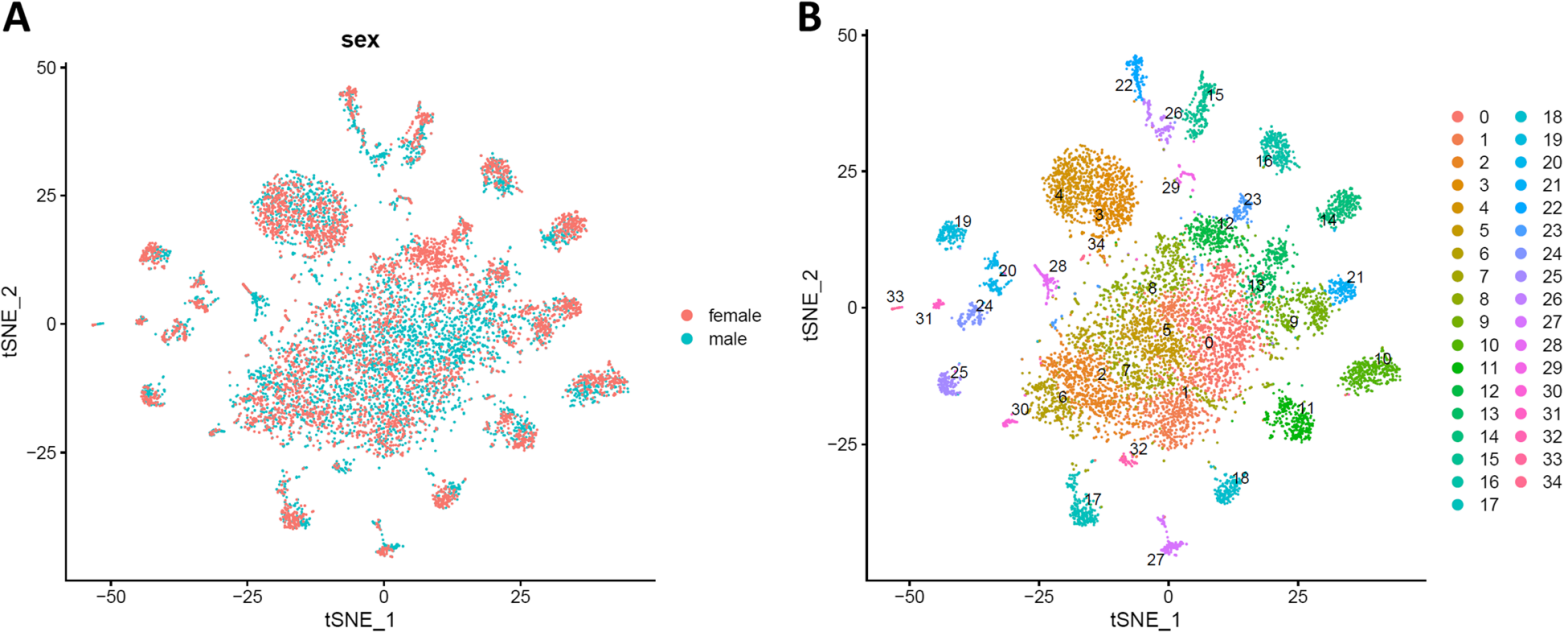
tSNE plots showing identified cell clusters in the brains of male and female *Ae. aegypti* mosquitoes. **A.** Two overlaid tSNE plots from the male (blue dots) and female (red dots) brain are shown. **B.** Thirty-five cell clusters shown in an integrated tSNE plot. The different cell clusters are color-coded. Each individual dot represents a single cell nucleus

We conducted a 10xChromium based snRNA-Seq experiment, which has recently been shown to be a reliable approach for the analysis of mosquito tissues [23]. Approximately 5000-6000 single nuclei from male and female brains, respectively, were used for the construction of two cDNA libraries. Low-quality nuclei with less than 500 or more than 4000 feature counts (unique genes) were filtered out (amounting to 10-15% of all nuclei) leaving 5356 nuclei from male brains and 4656 nuclei from female brains for the subsequent analyses (Fig. S1). A median number of 1295 (male brain) and 1628 (female brain) genes per nucleus were detected. In all male and female nuclei combined, 11,569 and 11,683 (absolute numbers) different protein-encoding genes, respectively were identified accounting for approximately 80% of the annotated genes (14,613 protein encoding genes) of the *Ae. aegypti* genome [24]. These data demonstrate that analyzing single nuclei of insect tissues instead of single cells is a reliable alternative method, which addresses the problem of the sample preparation of those tissues that cannot be readily dissociated into high-quality single-cell suspensions [25, 26]. Using Seurat v.3 [27, 28], the two snRNA-Seq datasets from male and female brains were integrated and analyzed. The t-distributed stochastic neighbor embedding (tSNE) plots based on non-linear dimension reduction [29] indicate that the nuclei of male and female brains contributed equally to the combined dataset (Fig. 1A). Using Seurat, 35 cell clusters expressing different marker genes were identified in the combined dataset (Fig. 1B, Table S1), and 15 clusters were assigned to known cell types mainly based on markers identified in the *Drosophila* brain [20–22]. We were unable to assign the remaining 20 clusters to any particular cell type and therefore designated these clusters with letters in alphabetical order from A to T (Table 1).

**Table 1:** Identification of cell clusters in the male and female brain of *Ae. aegypti*

Cluster	Cell type	Cellular proportion				Markers in ***Ae. aegypti***	Markers in ***Drosophila***
		Female	Male	Fold (F/M)	***p*** value		
0	Cluster A	0.0569	0.1286	0.44	***P*** **< 0.0001**		
1	Cluster B	0.0666	0.0723	0.92	*P* = 0.2638		
2	Cluster C	0.0674	0.0711	0.95	*P* = 0.4674		
3	**vPN 1**	0.0670	0.0450	1.49	***P*** **< 0.0001**	AAEL019970, AAEL005507, AAEL019457	*ct, acj6, Lim 1*
4	**vPN 2**	0.0655	0.0437	1.50	***P*** **< 0.0001**	AAEL019970, AAEL005507, AAEL019457	*ct, acj6, Lim 1*
5	Cluster D	0.0397	0.0620	0.64	***P*** **< 0.0001**		
6	Cluster E	0.0479	0.0541	0.88	*P* = 0.1604		
7	Cluster F	0.0279	0.0648	0.43	***P*** **< 0.0001**		
8	Cluster G	0.0412	0.0409	1.01	*P* = 0.9398		
9	Cluster H	0.0387	0.0330	1.17	*P* = 0.1250		
10	**KC 1**	0.0384	0.0327	1.18	*P* = 0.1235	AAEL002321, AAEL005834, AAEL019691	*eyeless, DopR2, sNPF*
11	**PN 1**	0.0341	0.0329	1.04	*P* = 0.7391	AAEL019970, AAEL005507	*ct, acj6*
12	Cluster I	0.0636	0.0056	11.35	***P*** **< 0.0001**		
13	Cluster J	0.0288	0.0357	0.81	*P* = 0.0521		
14	**KC 2**	0.0372	0.0217	1.72	***P*** **< 0.0001**	AAEL002321, AAEL005834, AAEL019977	*eyeless, DopR2, trio*
15	**glia 1**	0.0275	0.0260	1.06	*P* = 0.6424	AAEL027131, AAEL010145	*repo, nrv2*
16	**Mi1**	0.0284	0.0245	1.16	*P* = 0.2240	AAEL011643, AAEL007221	*hth, bsh*
17	Cluster K	0.0292	0.0185	1.58	***P*** **= 0.0004**		
18	Cluster L	0.0217	0.0157	1.38	***P*** **= 0.0262**		
19	Cluster M	0.0213	0.0129	1.65	***P*** **= 0.0011**		
20	Cluster N	0.0176	0.0151	1.16	*P* = 0.3239		
21	Cluster O	0.0249	0.0088	2.84	***P*** **< 0.0001**		
22	**astrocyte**	0.0165	0.0157	1.05	*P* = 0.7509	AAEL027131, AAEL007322, AAEL014510	*repo, wun2, sty*
23	**monoaminergic neuron**	0.0217	0.0099	2.19	***P*** **< 0.0001**	AAEL026276, AAEL024732, AAEL005581	*Vmat, DAT, SerT*
24	Cluster P	0.0150	0.0125	1.20	*P* = 0.2825		
25	Cluster Q	0.0152	0.0119	1.28	*P* = 0.1526		
26	**glia 2**	0.0032	0.0213	0.15	***P*** **< 0.0001**	AAEL027131, AAEL010145	*repo, nrv2*
27	Cluster R	0.0097	0.0153	0.63	***P*** **= 0.0126**		
28	Cluster S	0.0045	0.0162	0.28	***P*** **< 0.0001**		
29	**glia 3**	0.0067	0.0067	0.99	*P* = 1.0000	AAEL027131, AAEL010145	*repo, nrv2*
30	**Pm**	0.0028	0.0088	0.32	***P*** **= 0.0001**	AAEL011643, AAEL007120	*hth, Lim 3*
31	Cluster T	0.0060	0.0039	1.53	*P* = 0.1325		
32	**KC 3**	0.0017	0.0073	0.24	***P*** **< 0.0001**	AAEL002321, AAEL005834	*eyeless, DopR2*
33	**glia 4**	0.0032	0.0034	0.96	*P* = 0.8620	AAEL027131, AAEL010145	*repo, nrv2*
34	**vPN 3**	0.0021	0.0017	1.28	*P* = 0.6455	AAEL019970, AAEL005507, AAEL019457	*ct, acj6, Lim 1*

*KC* Kenyon cells, *Mi1* medulla neurons, *Pm* proximal medulla neurons, *PN* projection neurons, *vPN* ventral projection neurons, *Fold (F/M)* fold expression (female/male)

### Identification of the cell types in the male and female brain of *Ae. aegypti*

#### Glia cells and astrocytes

**Fig. 2:**
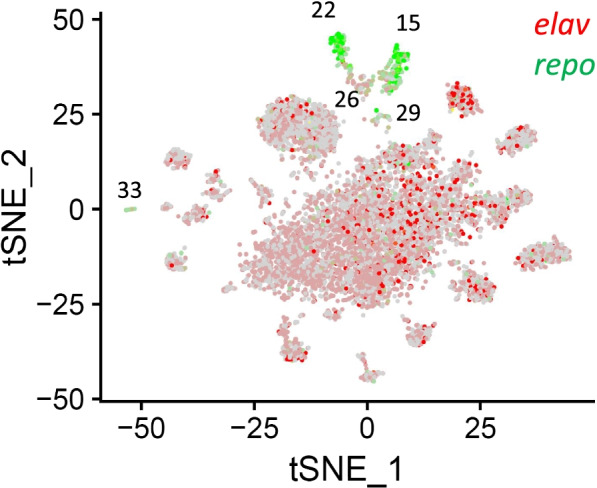
A tSNE plot showing the distribution of neurons and glia in the brain of *Ae. aegypti*. Neurons expressing the marker gene *elav* (AAEL008164) are in depicted in red, whereas glia expressing the marker gene *repo* (AAEL027131) are shown in green. Cell cluster numbers representing glia are indicated

Glia are non-neuronal cells in the central as well as in the peripheral nervous system that do not produce electrical impulses [30]. Until recently they were believed to play only a passive, supporting role. However, it has become obvious that these cells have additional, essential functions including making crucial contributions to the formation, operation, and adaptation of neural circuitry [30, 31]. In our study, five clusters (clusters 15, 22, 26, 29, and 33), which highly expressed a known glial marker, the homeobox transcription factor *reversed polarity* (*repo*) [32], but not the neuron marker *embryonic lethal abnormal visual system* (*elav*) [33], were assigned as glia cells (Fig. 2, Fig. S2, and Table 1). Among these five glia clusters, cluster 22 could be identified as astrocytes, a specialized type of glial cells, which highly co-expressed the astrocyte marker *wun2* (Fig. S2) [20]. Cell clusters 15, 26, 29, and 33 co-expressed multiple markers representing surface glia (*gemini*), ensheathing glia (*ebony*), and chiasm glia (*hoepel1*) analogous to their designations in *Drosophila*. Since we were not able to assign these clusters to a specific glia type, we designated them as glia #1, #2, #3, and #4. In total, 7.3 and 5.7% of the glia cells were detected in the male and female *Ae. aegypti* brain, respectively (Table 1).

#### Kenyon Cells (KC) in the mushroom bodies of the brain

The mushroom bodies of the insect brain are a pair of easily discernible neuropils consisting of three types of Kenyon cells (KC), α/β, α’/β’ and γ neurons, and are crucial for olfactory, visual, and gustatory learning [19, 34–36]. Three cell clusters (10, 14, and 32) highly expressed the two general KC markers *eyeless* and *DopR2* (Fig. S3) defining these cells as KC. However, the KC-type markers *short neuro-peptide F* (*sNPF*), *Fas2*, and *trio* described in *Drosophila* [20, 22] could not be specifically assigned to any of the three KC containing cell clusters, or any other cell clusters (Fig. S3). Consequently, we were not able to assign *Ae. aegypti* KC (sub)-types according to *sNPF*, *Fas2* and/or *trio* expression, and therefore designated them as KC #1, #2, and #3 (Table 1).

#### Olfactory Projection Neurons (PN)

Olfactory PN receive input from olfactory receptor neurons and glomeruli in the antennal lobes to carry olfactory information to higher brain centers [37]. Four clusters (3, 4, 11, and 34) co-expressed the two olfactory PN markers *cut* and *abnormal chemosensory jump* 6 (*acj6*) identified in *Drosophila* [20] and therefore were identified as PNs (Table 1, Fig. S4). Since cell clusters 3, 4, and 34 also expressed the ventral PN (vPN) marker *Lim 1*, we further specified these as vPN #1, #2, and #3, respectively.

#### Optic lobe neurons

Cell cluster 16 co-expressed both medulla neuron markers, *homothorax* (*hth)* and *brain-specific homebox* (*Bsh*), and therefore was assigned as Mi1 medulla neurons [21, 38, 39] (Table 1, Fig. S5). Cluster 30 highly expressed both, *hth*, and *Lim 3* indicative of a proximal medulla (Pm) cell type based on studies in *Drosophila*. Since no other markers with similarities to known *Drosophila* Pm cells were highly expressed, we did not further specify the Pm cell type of cluster 30.

#### Neurotransmitters

**Fig. 3:**
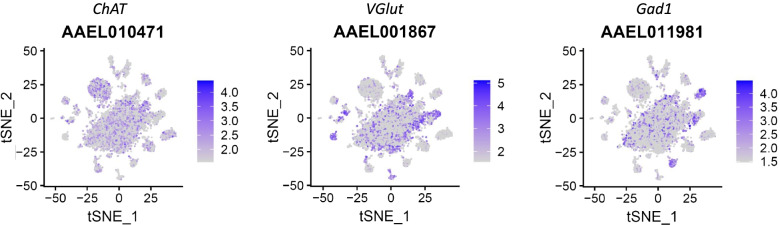
Distribution of fast-acting neurotransmitters among the brain cells of *Ae. aegypti*. tSNE plots showing the distribution of brain cells expressing *choline acetyltransferase* (*ChAT*, cholinergic neurons), *vesicular glutamate transporter* (*VGlut*, glutamatergic neurons), and *glutamic acid decarboxylase 1* (*Gad1*, GABA-ergic neurons)

Insects possess three major neurotransmitters, acetylcholine, vesicular glutamate transporter, and GABA [8]. Acetylcholine is the primary excitatory neurotransmitter in the central nervous system, whereas glutamate is used for neuromuscular transmission from motor neurons to muscles, and GABA is generally considered to be the primary inhibitory neurotransmitter in insect central synapses. The cells expressing choline acetyltransferase (*ChAT*), vesicular glutamate transporter (*VGlut*) and glutamic acid decarboxylase 1 (*Gad1*) are cholinergic, glutamatergic, or GABA-ergic neurons [40, 41]. These three markers were identified in different brain cell clusters of *Ae. aegypti* or among different cells within the same cluster (Fig. 3), which is consistent with the expression patterns of the neurotransmitters in other insects including *Drosophila* [20].

#### Monoaminergic neurons

**Fig. 4:**
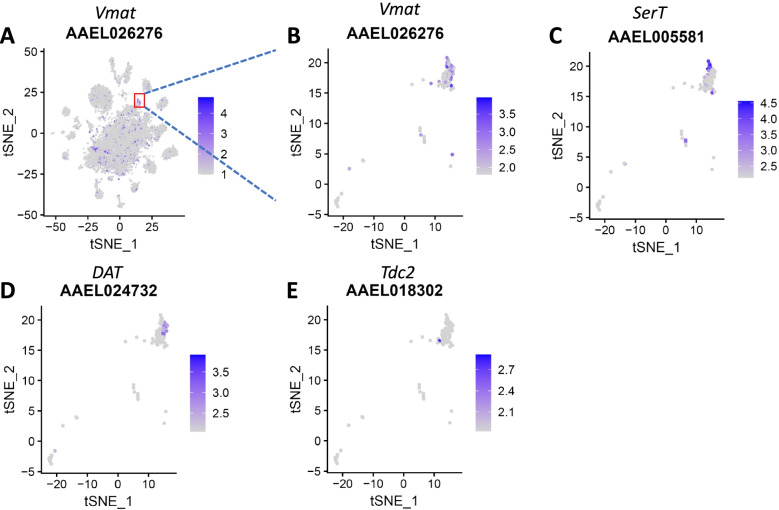
Different neuron types identified in brain cell cluster 23 of *Ae. aegypti*. **A, B** tSNE plots showing the distribution of brain cells in cluster 23 expressing the vesicular transporter gene, *Vmat* indicative of monoaminergic neurons. **C-E** Within cluster 23, other cells expressing the markers *serotonin transporter* (*SerT*), *dopamine transporter* (*DAT*), and *Tdc2* were identified, representing serotonergic, dopaminergic, and octopaminergic/tyraminergic neurons, respectively

Monoaminergic neurons are essential components of all nervous systems across the animal kingdom [42]. Their terminally differentiated state is defined by the coordinated expression of specific enzymes and transporters that synthesize a specific monoamine. Cluster 23 highly expressed the vesicular transporter-encoding gene, *Vmat*, indicative of monoaminergic neurons (Fig. 4A,B, Table 1). Furthermore, serotonergic cells expressing *serotonin transporter* (*SerT*) and dopaminergic neurons (DA) expressing *dopamine transporter* (*DAT*), as well as octopaminergic/tyraminergic neurons expressing *Tdc2* were identified in this cluster (Fig. 4C-E). The presence of these monoaminergic neurons in the mosquito brain suggests that they, similar to the situation in *Drosophila*, might control reward, aggression, oviposition choice, and social behavior in mosquitoes [43, 44].

### Cellular compositions of the male and female mosquito brains are different

When comparing the proportions of each cell type in the brain of males and females, significant differences in 17 out of the 35 cell clusters became apparent. Proportionally, nine cell clusters including clusters 3 (PN #1), 4 (PN #2), 12, 14 (KC #2), 17, 18, 19, 21, and 23 (DA) were significantly more abundant (*p* value < 0.05) in the female brain than in the male brain. By contrast, eight cell clusters including clusters 0, 5, 7, 26 (glia #2), 27, 28, 30 (Pm), and 32 (KC #3) were significantly more abundant in the male brain than in the female brain (Table 1). Differences in the cellular compositions, especially in KC, DA, and PN cells suggest that there are also differences in the size and structure of the brain components containing these cells between males and females underlying their sexually dimorphic behaviors [19].

### The transcriptomes of male and female brain cell clusters are highly-similar and contain only a few differentially expressed genes

**Fig. 5:**
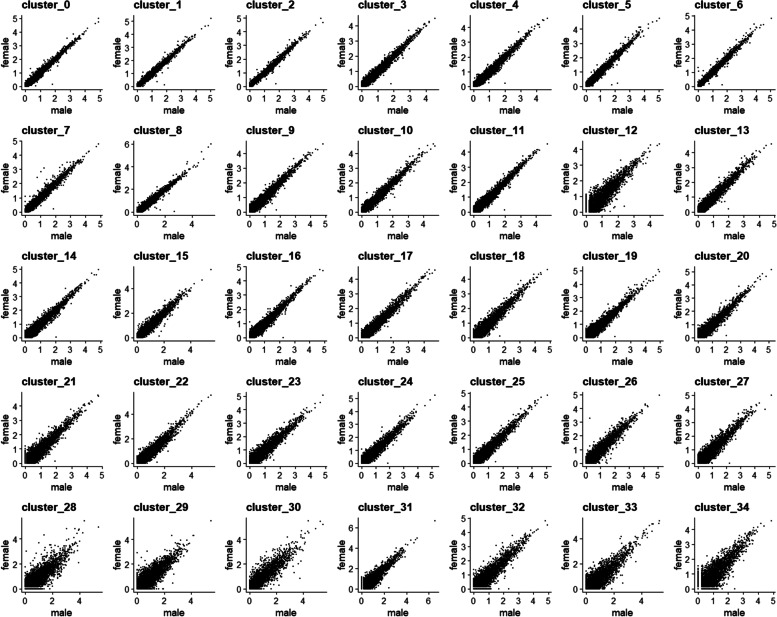
Comparison of the transcriptomes of each cell type of the male and female *Ae. aegypti* brain. The average expression level of each gene in each cell cluster was compared between the male (x-axis) and female (y-axis) brain. Each dot represents the average expression level of a single gene. Dots along the 45-degree angle line represent genes whose expression levels did not differ significantly between the male and female brain

For the majority of the various cell types, gene expression profiles were very similar between the male and female brain (Fig. 5) as only up to 25 differentially expressed genes per cluster were found among the 35 identified brain cell clusters (fold change of female/male ≥2 or ≤ 0.5 and *p* value < 0.05) (Table S2). Nevertheless, a few genes including *Nix*, *dipeptidyl-peptidase*, and *insulin-like peptide 7* (*ILP 7*) were highly-differentially expressed in the male and female brains.

#### *Nix* is highly expressed in neurons and glia of the male brain

**Fig. 6:**
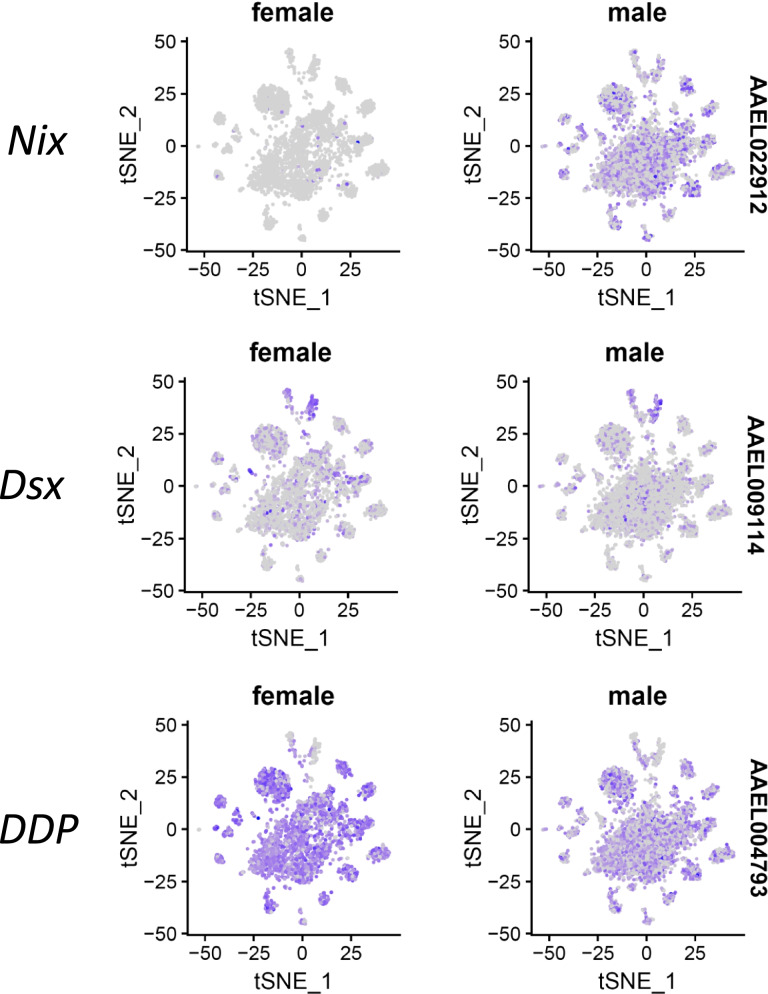
tSNE plots showing side-by-side the distribution of *Nix* (AAEL022912), *doublesex* (*dsx*; AAEL009114), and *dipeptidyl-peptidase* (*DDP*; AAEL004793) expressing cells in the male and female brain of *Ae. aegypti*

Sex determination in the mosquito *Ae. aegypti* is governed by a dominant male-determining factor (M factor) located within a Y chromosome–like region called the M locus [45]. *Nix*, which is located within the M-locus and functions as an M factor, is required and sufficient to initiate male development in *Ae. aegypti* and *Ae. albopictus* [46–48]. In our study, *Nix* was highly and widely expressed in the neurons and glia of the male brain (Fig. 6). We also detected *Nix* expression in very few cells of the female brain (Fig. 6). In earlier RNA-Seq studies, *Nix* was also detected in the brain, antennae, and ovaries of female *Ae. aegypti* mosquitoes albeit at very low levels (background noise) [8, 49, 50].

#### *Dsx* is expressed in neurons and glia of the male and female brain

The dsx proteins are critically important for sex determination throughout the animal kingdom. In *Ae. aegypti*, *dsx* is sex-specifically regulated and encodes two female-specific and one male-specific isoforms [51]. We observed that *dsx* was expressed in all cell clusters of the male and female brain and its expression level was much higher in glia than in neurons (Fig. 6). Since the 10xChromium platform analyzes only 3′ ends of mRNAs, we could not obtain any information about potential isoforms of the sequenced transcripts. However, we infer that the male-specific *dsx* isoform is restricted to the male brain while the female-specific *dsx* isoforms are detected in the female brain. In *Drosophila*, Rideout et al. [52] observed that *dsx*-expressing cells not only reprise the functional roles of endogenous dsx in establishing external sexual morphology, but also establish a dimorphic neuro-anatomy capable of directing distinct sex-specific behavioral outputs. Thus, the wide expression of *dsx* in the male and female *Ae. aegypti* brain implies that *dsx*-expressing neurons and glia direct sexually-dimorphic behaviors in mosquitoes.

#### *Dipeptidyl-peptidase* is highly expressed in the female brain

Expression levels of *dipeptidyl-peptidase* were significantly higher in the female brain of *Ae. aegypti* in comparison to the male brain of the mosquito as shown in 20 of the 35 cell clusters (Fig. 6). Recently, higher levels of *dipeptidyl-peptidase* expression were observed in the female antennae of *Ae. aegypti* than in the antennae of males [50] indicating an involvement of the peptidase in olfaction and perhaps mating behavior [53]. The differential expression of *dipeptidyl-peptidase* in neurons and glia in the male and female mosquito brain suggests that *dipeptidyl-peptidase* expressing cells affect sexually-dimorphic behaviors in mosquitoes.

#### ILP 7 expression in the male and female brain

ILP 7 is one of the brain-specific ILP in *Ae. aegypti* [54]. Depletion of *ilp7* leads to increased triacylglyceride (TAG) and decreased glycogen synthesis [55]. We observed that the expression of *ilp7* in the brain cells belonging to cluster 12 was significantly lower in sugarfed females than in (sugarfed) males (Table S2). A low *ilp7* expression level in females may lead to increased TAG synthesis, which then could provide the energy required for host seeking and reproduction.

## Discussion

Here we present for the first time a transcriptomic analysis of the male and female brain of *Ae. aegypti* at the single-nucleus level. Initially, we tried to isolate single (whole) brain cells for the transcriptomic analysis (scRNA-Seq) but cell viability proved to be insufficient. In a previous transcriptome analysis, we showed that focusing on nuclear mRNA only instead of using cytoplasmic and nuclear mRNA from midgut cells of mosquitoes resulted in high-quality data with sufficient resolution [23]. Thus, we decided to use snRNA-Seq for our brain cell transcriptome analysis. As a result, a cell atlas of the male/female mosquito brain comprising of 35 different cell types was generated from ~ 5000 single nuclei obtained from male and female brain cells, respectively. By comparison, 87 cell clusters had been identified from 56,902 cells (26 runs) isolated from the whole brain of *Drosophila* [22]. Thus, we identified less than half as many cell clusters from the brain of *Ae. aegypti* as have been described for the *Drosophila* brain and our relatively small sample size representing a single replicate could be a reason for this. Regardless, our cell cluster assignment strategy for the mosquito brain followed the strategy applied for the *Drosophila* brain [20–22]. We noticed that most of the potential brain cell markers in *Ae. aegypti*, similar to *Drosophila*, were not unique for a specific cell cluster and therefore the expression pattern of a combination of genes had to be taken into consideration in order to distinguish cell types from each other. Furthermore, the overall sequencing depth of our snRNA-Seq analysis of the *Ae. aegypti* brain reflected that of the scRNA-Seq analysis of the *Drosophila* brain [22]. In our analysis, a median number of 1295 (male brain) and 1628 (female brain) genes per nucleus were detected, while in the scRNA-Seq analysis of the *Drosophila* brain, 1308 genes per cell at 0 day and 810 genes per cell at day 50 were identified [22].

Given that there are ~ 248,000 cells in the brain of *Ae. aegypti* [9], with no statistical difference in the total number of brain cells between males and females, our study only covers ~ 2% of the cells present in the mosquito brain. It needs to be clearly pointed out that our experimental design consisted of only a single biological replicate. Thus, a further validation of our data was hampered by the lack of replicates, allowing the possibility that some of our results and findings may be subject to change in any follow-up studies. For example, it is conceivable that some rare brain cell types have not been detected in our analysis. A follow-up analysis of the mosquito brain should ideally include several biological replicates, different developmental stages, ages, and perhaps even another mosquito species for comparison. As there are thousands of transgenic GAL4 lines of *Drosophila* available that direct expression in specific subsets of neurons in the brain, these resources present currently the most straightforward means to link single-cell sequencing data to neuro-anatomy thereby simplifying the annotation of brain cell types in *Drosophila* [20, 56, 57]. Regardless, even with these powerful resources at hand, a substantial number of cell clusters from the midbrain, optic lobes, or whole brain has still not been annotated in *Drosophila*, exemplifying the current limitations of understanding the structure, physiology, and function of the insect brain. In our study, we were able to assign 15 out of 35 cell clusters to a particular cell type mainly based on markers for neurons and glia from *Drosophila*. The number of 35 brain cell clusters for *Ae. aegypti* may be an under- or over-estimate of the actual number of cell clusters present in the mosquito brain. Some of the cell clusters such as clusters 0, 1, and 2 appeared to be fairly large. However, we did not perform any further sub-clustering since we were not able to assign any cell type designations to these clusters. Nonetheless, our study represents a useful starting point for a more complete cell type annotation in the future, which could include the use of neuron cell type-specific transgenic mosquito lines for brain cell annotation and functional studies. Recently, Matthews et al. [58] generated a sensory neuron specific *Ae. aegypti* line expressing ppk301, while Jové et al. [59] generated a female stylet neuron-specific driver line and Zhao et al. [60] a pan-neuronal genetic driver line in *Ae. aegypti*. However, so far there is no report about a neuron cell type-specific driver, which perhaps could be developed in the future utilizing the cell type markers we have identified in this study (Table 1) as well as potential markers listed in Table S1.

The relative abundance of glia cells in the brains of male and female *Ae. aegypti* was remarkably close to that of glia in the *Drosophila* brain (6.4%) [22], and it is only slightly lower than the relative abundance of non-neuronal cells in the brain of *Ae. aegypti* as revealed when using an isotropic fractionator coupled with immunohistochemistry [9]. This indicates that these two dipteran insects have similar numbers (relative quantities) of neurons and glia in their brains despite their evolutionary distinct developments [61]. We identified the markers *choline acetyltransferase* (*ChAT*), *vesicular glutamate transporter* (*VGlut*), and *glutamic acid decarboxylase 1* (*Gad1*) among several cell clusters of the male and female mosquito brain. Other important neurotransmitters/modulators in *Ae. aegypti* include serotonin, dopamine, tyramine, octopamine, and histamines [8, 62], which we could not assign to specific cell types in our study. In *Drosophila*, behavioral processes such as reward, aggression, oviposition choice, and social behavior have been implicated to be controlled by these amine neurotransmitters [43, 44]. In *Ae. aegypti*, expression of serotonin and dopamine has been observed in the brain, antennal lobes, and other peripheral tissues (i.e., midgut and legs) [8].

The sexually-dimorphic behaviors of male and female mosquitoes might be reflected by their sex-specific brain cell type compositions and/or neuro gene expression patterns. Accordingly, we observed highly-distinct cellular compositions in the male and female brain of *Ae. aegypti.* By contrast, the overall gene expression patterns within each cell cluster proved to be very similar between the male and female brains. We found that *Nix* was highly expressed in neurons and glia of the male brain while *dsx* was widely expressed in cell clusters of the male and female brain. In *Drosophila*, *dsx* is essential for the sexual development of both neuronal and non-neuronal tissues, thereby establishing external sexual morphology, as well as a dimorphic neuroanatomy capable of directing distinct sex-specific behaviors [52]. Specifically, *dsx*-expressing neurons in the *Drosophila* brain receive sex-specific sensory information required for courtship pursuit in males and olfactory signals required for oviposition in females [63]. Accordingly, the neurons highly expressing *Nix* and *dsx* might play important roles in sexually-dimorphic behaviors of mosquitoes, which remains to be further investigated. Another sex-related gene was *dipeptidyl-peptidase*, which was significantly overexpressed in 20 out of 35 clusters of the female brain when compared to the male brain. In *Drosophila*, *dipeptidyl-peptidase* expression levels were higher in the female antennae than in the antennae of males [50] indicating an involvement of the peptidase in olfaction and perhaps mating behavior [53]. Understanding the molecular mechanism underlying *dipeptidyl-peptidase* upregulation in mosquitoes requires further investigation.

## Conclusions

We generated an initial cell atlas of the *Ae. aegypti* brain using 10xGenomics based snRNA-Seq and identified 35 cell clusters from male and female brains. We revealed that a major distinction between the brains of the two sexes is their relative cell type composition, whereas gene expression patterns between the matching clusters of the male and female brains were very similar. The male sex determination factor, *Nix*, was highly expressed in the neurons and glia of the male brain, whereas *dsx* was widely expressed in neurons and glia of the male and female brain. Our snRNA-Seq study based on a single biological replicate provides a resource for further studies on the biology of the mosquito brain and sexually-dimorphic behaviors of mosquitoes.

## Methods

### Mosquitoes

Mosquitoes were reared in an insectary at 28^o^ C, 80% relative humidity, and under a 12 h light/12 h dark cycle. Hatched larvae of *Ae. aegypti* (strain: Liverpool strain, LVP) were maintained in plastic shoe boxes (100 larvae/box) each filled with 500 ml of distilled water and fed with tropical fish food (Tetramin, Melle, Germany). Male and female pupae were separated and placed in groups of 100 individuals in small (1 oz.) cups filled with distilled water. Each pupa cup was placed into a separate ice cream carton (64 oz.) covered by an organdy. Adults were fed *ad libitum* on raisins until further analysis.

### Single nuclei isolations from mosquito brains

Thirty brains were dissected from 7-day old adult *Ae. aegypti* (LVP) males and females, respectively, and placed in Schneider’s *Drosophila* medium on ice. The brain tissue was minced thoroughly on a pre-chilled petri-dish and then homogenized using micro pestle (Sigma) for 30 s. The samples were resuspended in Schneider’s Drosophila medium and centrifuged for 5 min at 500x *g* and 4^o^ C. Following aspiration of the supernatant, single nucleus isolation was performed using the Pure Prep Nuclei Isolation kit (Sigma, St. Louis, MO, USA). Briefly, the cell nuclei containing pellet was resuspended in 100 μl of fresh lysis buffer [Nuclei PURE Lysis Buffer containing 1 mM dithiothreitol (DTT)] and homogenized using a micro-pestle before another 400 μl of lysis buffer was added. The nuclei suspension was then incubated for 15 min on ice before 900 μl of 1.8 M sucrose cushion buffer was added to the resuspension. After mixing, 700 μl of the solution was carefully placed onto a 1.8 M sucrose cushion (500 μl v/v) in a 1.5 ml microcentrifuge tube. Following centrifugation at 13,000x *g* and 4^o^ C for 45 min, the resulting supernatant was gently aspirated without disturbing the pellet. The pellet then was resuspended in 500 μl of Nuclei PURE Storage Buffer. The resuspension was filtered using a 40 μm cell strainer (Fisher Scientific) before the filtered resuspension was centrifuged at 500x *g* and 4^o^ C for 5 min. Again, the supernatant was aspirated and then resuspended in 200 μl of storage buffer. Cell nuclei were stained with trypan blue, and their concentration was determined using a Countess II FL Automated Cell Counter (ThermoFisher).

### Single-nucleus RNA sequencing (snRNA-Seq) using the 10xChromium platform

The 10xChromium platform was used to sequence total RNA from nuclei of single cells dissected from male and female brains. The sequencing libraries were prepared using the Chromium Next GEM Single Cell 3′ GEM, Library & Gel Bead Kit v3.1 at the University of Missouri DNA Core facility following the manufacturer’s instructions. We targeted to sequence 5000 single nuclei at a depth of 20,000 paired-end (single-indexing) reads per nuclei. The base call (BCL) files generated from the Illumina sequencing were processed by Cell Ranger (v. 3.0.1) and converted to FASTQ files by the ‘mkfastq’ function. The reads were mapped to the *Ae. aegypti* reference genome Aaegl5 using STAR aligner [64] with the default setting of the Cell Ranger pipeline. The Cell Ranger pipeline was used to perform background filtration based on UMI (unique molecular identities) versus barcode counts, and to generate feature-barcode matrices from the libraries. The ‘count’ function of Cell Ranger was used to count barcodes and UMI and to generate the read counts of all features (genes) in individual cells. Using the R package Seurat [27], the read count data of genes of single cells was analyzed with the help of the ‘Read10x’ function. Any low-quality nuclei with < 500 or > 4000 feature counts were filtered out and the remaining nuclei were used for sequential analysis. Data normalization was performed using the ‘LogNormalize’ method implemented within Seurat. Using the ‘FindVariableFeatures’ function, the normalized data was then screened to identify the genes with variable expression levels across all cells. We integrated the gene expression data of both sexes by identifying integration anchors for the first 20 dimensions of data variation by canonical correlation analysis (CCA) [27]. The anchors were then used to integrate the expression data of male and female brains using the ‘IntegrateData’ function. This normalized and integrated data was then subjected to principal component analysis (PCA) applying t-distributed stochastic neighbor embedding (tSNE) to identify individual clusters of cells in the brain of both sexes [29]. The ‘FindAllMarkers’ function was used to identify the marker genes that showed significantly differential expression in each cluster. The identified maker genes were used to annotate cell types based on *Drosophila* brain data [20, 22]. All the raw and processed data from this study are available in the Gene Expression Omnibus (GEO) database under the accession number GSE160740.

### Comparative gene expression analyses of male and female brain cell clusters

The cellular compositions of male and female brains were compared and the Chi-square test was used for statistical analysis. The average expression levels of the genes detected from each male and female brain cell cluster were plotted on a scatter plot. Differentially expressed genes were identified in each cell cluster based on fold-change between male/female brains ≥2 or ≤ 0.5 and a *p*-value < 0.05.

## Supplementary Information

**Additional file 1: Table S1.** Identification of the marker genes for the brain cells of *Aedes aegypti*.

**Additional file 2: Table S2.** Differentially expressed genes in each cell cluster of the male and female brain of *Aedes aegypti*.

**Additional file 3: Figure S1.** Features (unique genes), counts (total # of RNA molecules), and abundance of mitochondrial RNA identified in each cell nucleus of the (**A**) male and (**B**) female brain preparations from *Aedes aegypti* mosquitoes. Each brain preparation consisted of a pool of ~ 30 brains. **Figure S2.** tSNE plots showing co-expression of glial cell markers in the brain of *Aedes aegypti*. Glia expressing the common marker *repo* (AAEL027131) are depicted in red and those glia expressing the specific glia cell markers *wun2* (AAEL007322), *gemini* (AAEL007800), *ebony* (AAEL005793), or *hoepel1* (AAEL007979) are shown in green. Cell cluster numbers representing glia are indicated. **Figure S3.** tSNE plots showing Kenyon cell markers in the brain of *Aedes aegypti*. Kenyon cells co-expressing the two general markers *eyeless* (AAEL002321) and *DopR2* (AAEL005834) are shown in red and green, respectively. Kenyon cells expressing the specific markers *sNPF* (AAEL019691), *Fas2* (AAEL009173), and *trio* (AAEL019977) are shown in green. Cell cluster numbers representing Kenyon cells are indicated. **Figure S4.** tSNE plots showing olfactory projection neurons (PN) in the brain of *Aedes aegypti*. The PNs co-express the two general markers *cut* (AAEL019970; shown in red) and *acj6* (AAEL005507; shown in green), whereas ventral (v) PNs also co-express the marker gene *Lim 1* (AAEL019457; in green). Cell cluster numbers representing PN/vPN are indicated. **Figure S5.** tSNE plots showing Mi1 medulla and proximal medulla (Pm) neurons in the optic lobes of the brain of *Aedes aegypti*. Mi1 neurons co-express the marker genes *bsh* (AAEL007221; in red) and *hth* (AAEL011643; in green), whereas Pm neurons co-express *hth* (in red) and *Lim 3* (AAEL007120; in green). Cell cluster numbers representing Mi1 and Pm neurons are indicated.

## Data Availability

All the raw and processed data from this study are available in the Gene Expression Omnibus (GEO) database under the accession number GSE160740 (https://www.ncbi.nlm.nih.gov/geo/query/acc.cgi?acc=GSE160740). The mosquitoes of the Liverpool (LVP) strain have been obtained in 2013 from Colorado State University, Fort Collins, CO, and since then they have been maintained in the corresponding author’s laboratory at the University of Missouri.
