# Supplementary material for: Cellular diversity and gene expression profiles in the male and female brain of Aedes aegypti
Source: BMC Genomics. 2022 Feb 10;23:119. doi: 10.1186/s12864-022-08327-9 (PMC8832747; doi:10.1186/s12864-022-08327-9)
Supplement: Supplementary file 3 — Additional file 3: Figure S1. Features (unique genes), counts (total # of RNA molecules), and abundance of mitochondrial RNA identified in each cell nucleus of the (A) male and (B) female brain preparations from Aedes aegypti mosquitoes. Each brain preparation consisted of a pool of ~ 30 brains. Figure S2. tSNE plots showing co-expression of glial cell markers in the brain of Aedes aegypti. Glia expressing the common marker repo (AAEL027131) are depicted in red and those glia expressing the specific glia cell markers wun2 (AAEL007322), gemini (AAEL007800), ebony (AAEL005793), or hoepel1 (AAEL007979) are shown in green. Cell cluster numbers representing glia are indicated. Figure S3. tSNE plots showing Kenyon cell markers in the brain of Aedes aegypti. Kenyon cells co-expressing the two general markers eyeless (AAEL002321) and DopR2 (AAEL005834) are shown in red and green, respectively. Kenyon cells expressing the specific markers sNPF (AAEL019691), Fas2 (AAEL009173), and trio (AAEL019977) are shown in green. Cell cluster numbers representing Kenyon cells are indicated. Figure S4. tSNE plots showing olfactory projection neurons (PN) in the brain of Aedes aegypti. The PNs co-express the two general markers cut (AAEL019970; shown in red) and acj6 (AAEL005507; shown in green), whereas ventral (v) PNs also co-express the marker gene Lim 1 (AAEL019457; in green). Cell cluster numbers representing PN/vPN are indicated. Figure S5. tSNE plots showing Mi1 medulla and proximal medulla (Pm) neurons in the optic lobes of the brain of Aedes aegypti. Mi1 neurons co-express the marker genes bsh (AAEL007221; in red) and hth (AAEL011643; in green), whereas Pm neurons co-express hth (in red) and Lim 3 (AAEL007120; in green). Cell cluster numbers representing Mi1 and Pm neurons are indicated. [file 12864_2022_8327_MOESM3_ESM.pptx]

## Slide 1
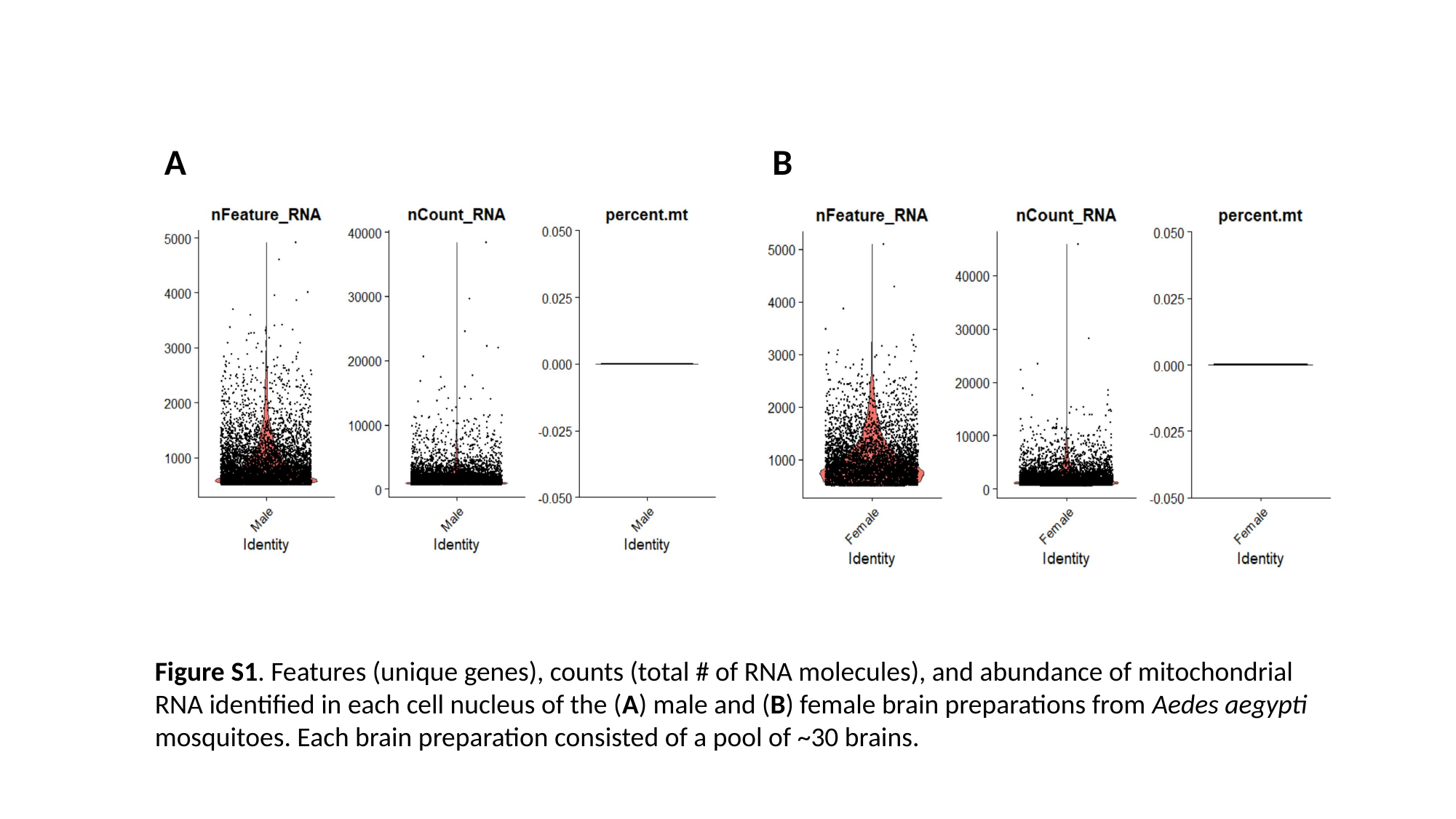

A
B
Figure S1. Features (unique genes), counts (total # of RNA molecules), and abundance of mitochondrial RNA identified in each cell nucleus of the (A) male and (B) female brain preparations from Aedes aegypti mosquitoes. Each brain preparation consisted of a pool of ~30 brains.

## Slide 2
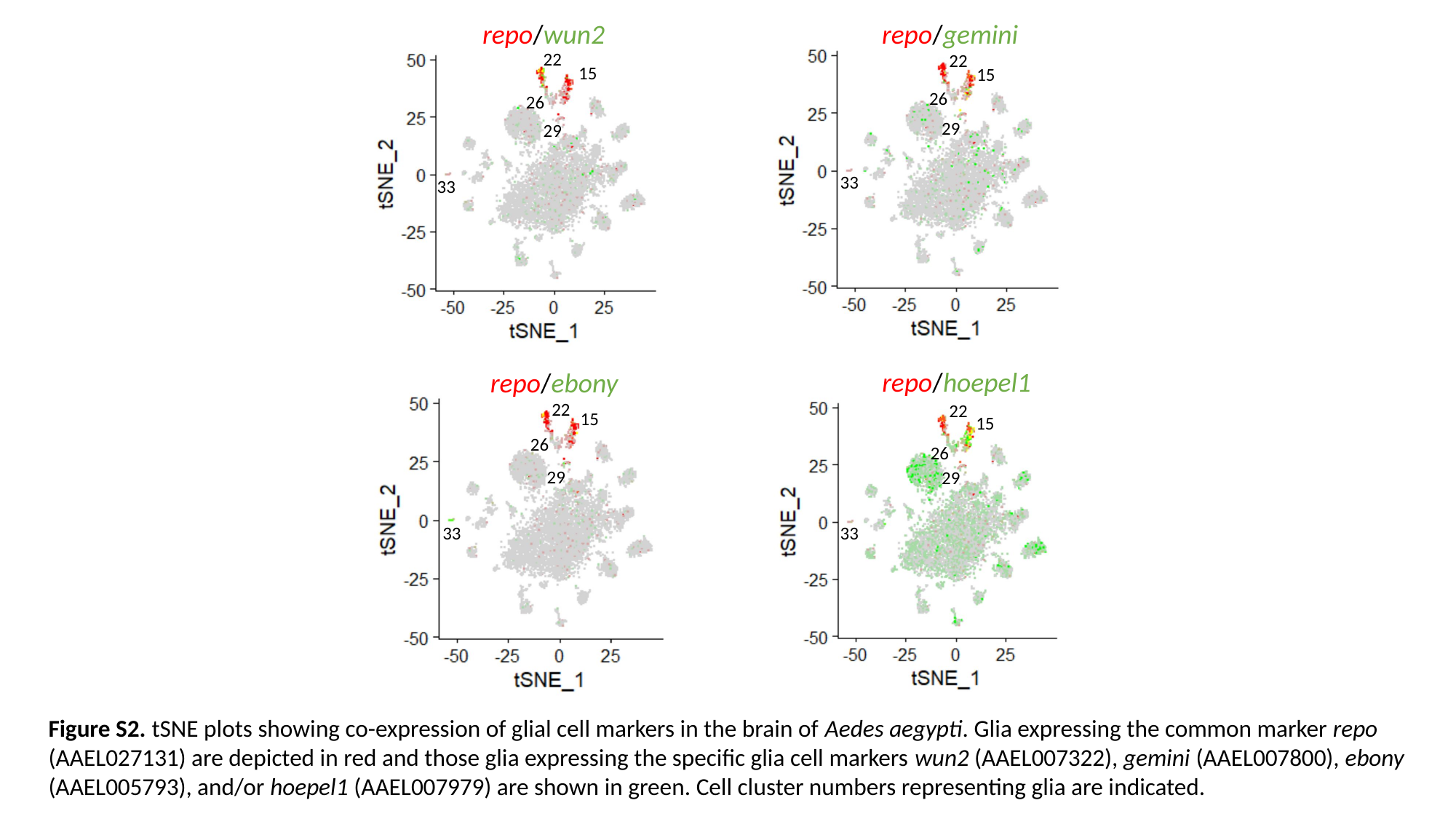

repo/wun2
22
repo/gemini
repo/hoepel1
repo/ebony
22
15
15
26
26
29
29
33
33
22
22
15
15
26
26
29
29
33
33
Figure S2. tSNE plots showing co-expression of glial cell markers in the brain of Aedes aegypti. Glia expressing the common marker repo (AAEL027131) are depicted in red and those glia expressing the specific glia cell markers wun2 (AAEL007322), gemini (AAEL007800), ebony (AAEL005793), and/or hoepel1 (AAEL007979) are shown in green. Cell cluster numbers representing glia are indicated.

## Slide 3
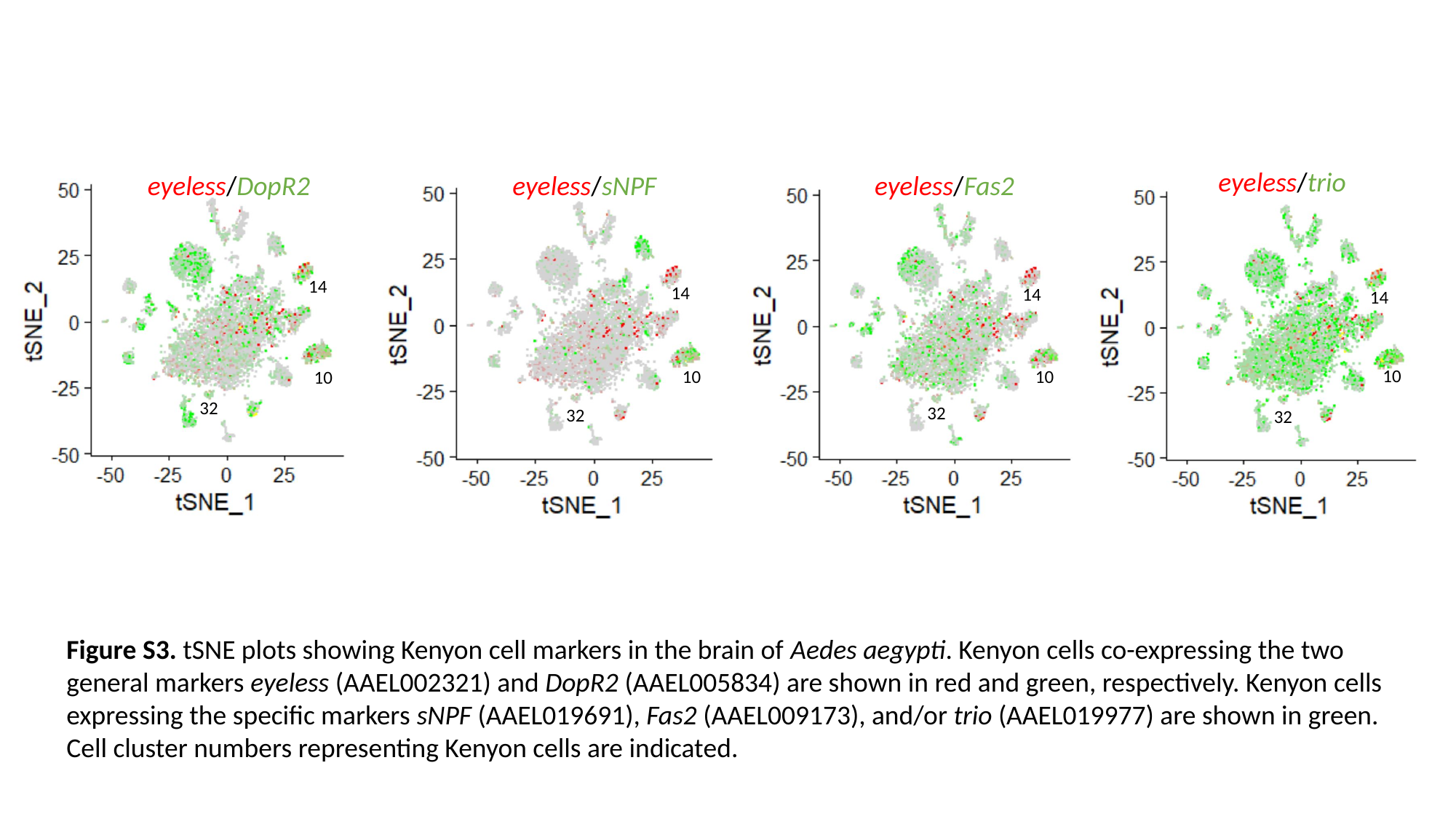

eyeless/trio
eyeless/DopR2
14
10
32
eyeless/sNPF
eyeless/Fas2
14
14
14
10
10
10
32
32
32
Figure S3. tSNE plots showing Kenyon cell markers in the brain of Aedes aegypti. Kenyon cells co-expressing the two general markers eyeless (AAEL002321) and DopR2 (AAEL005834) are shown in red and green, respectively. Kenyon cells expressing the specific markers sNPF (AAEL019691), Fas2 (AAEL009173), and/or trio (AAEL019977) are shown in green. Cell cluster numbers representing Kenyon cells are indicated.

## Slide 4
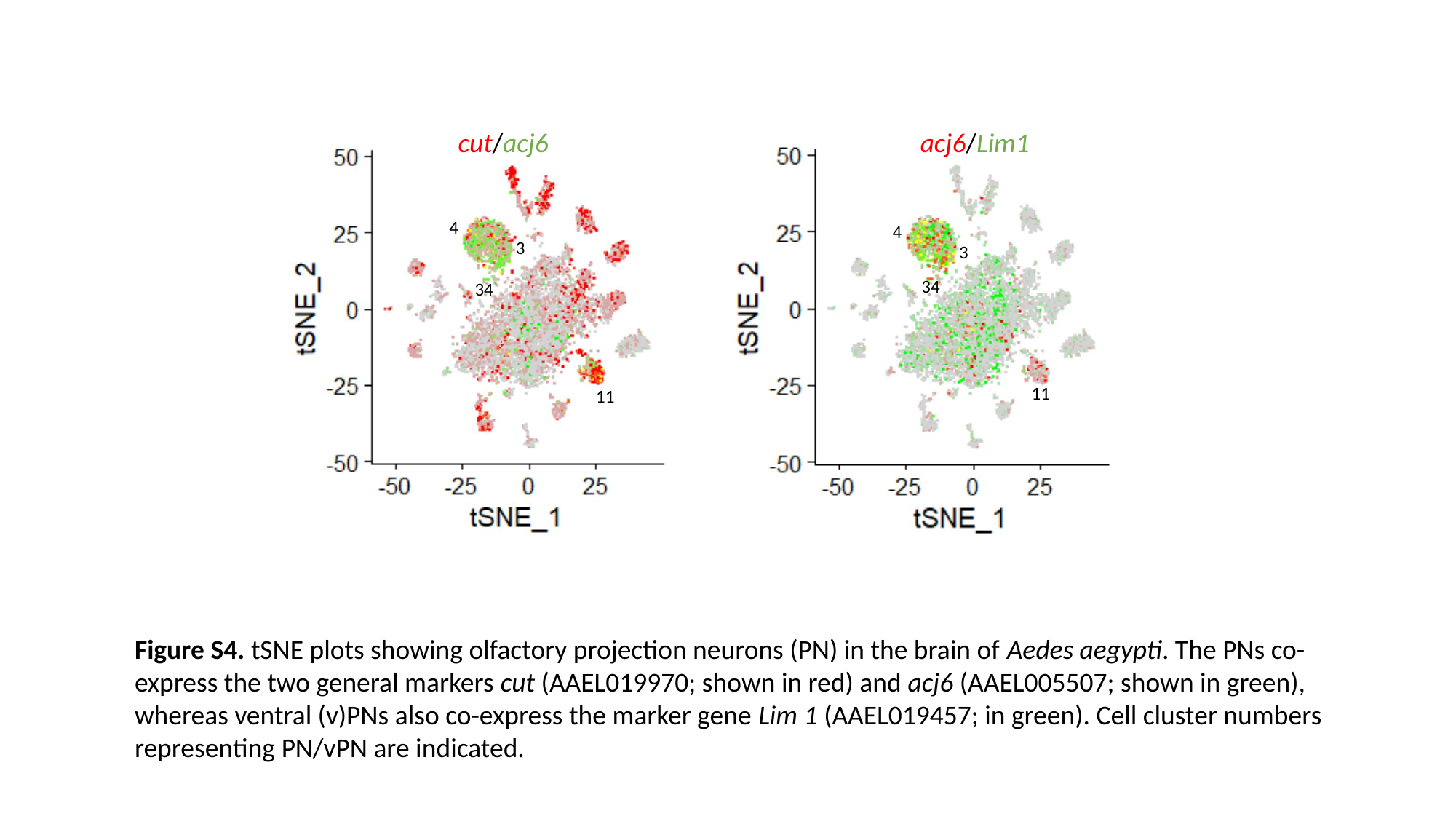

acj6/Lim1
4
3
34
cut/acj6
4
3
34
11
11
Figure S4. tSNE plots showing olfactory projection neurons (PN) in the brain of Aedes aegypti. The PNs co-express the two general markers cut (AAEL019970; shown in red) and acj6 (AAEL005507; shown in green), whereas ventral (v)PNs also co-express the marker gene Lim 1 (AAEL019457; in green). Cell cluster numbers representing PN/vPN are indicated.

## Slide 5
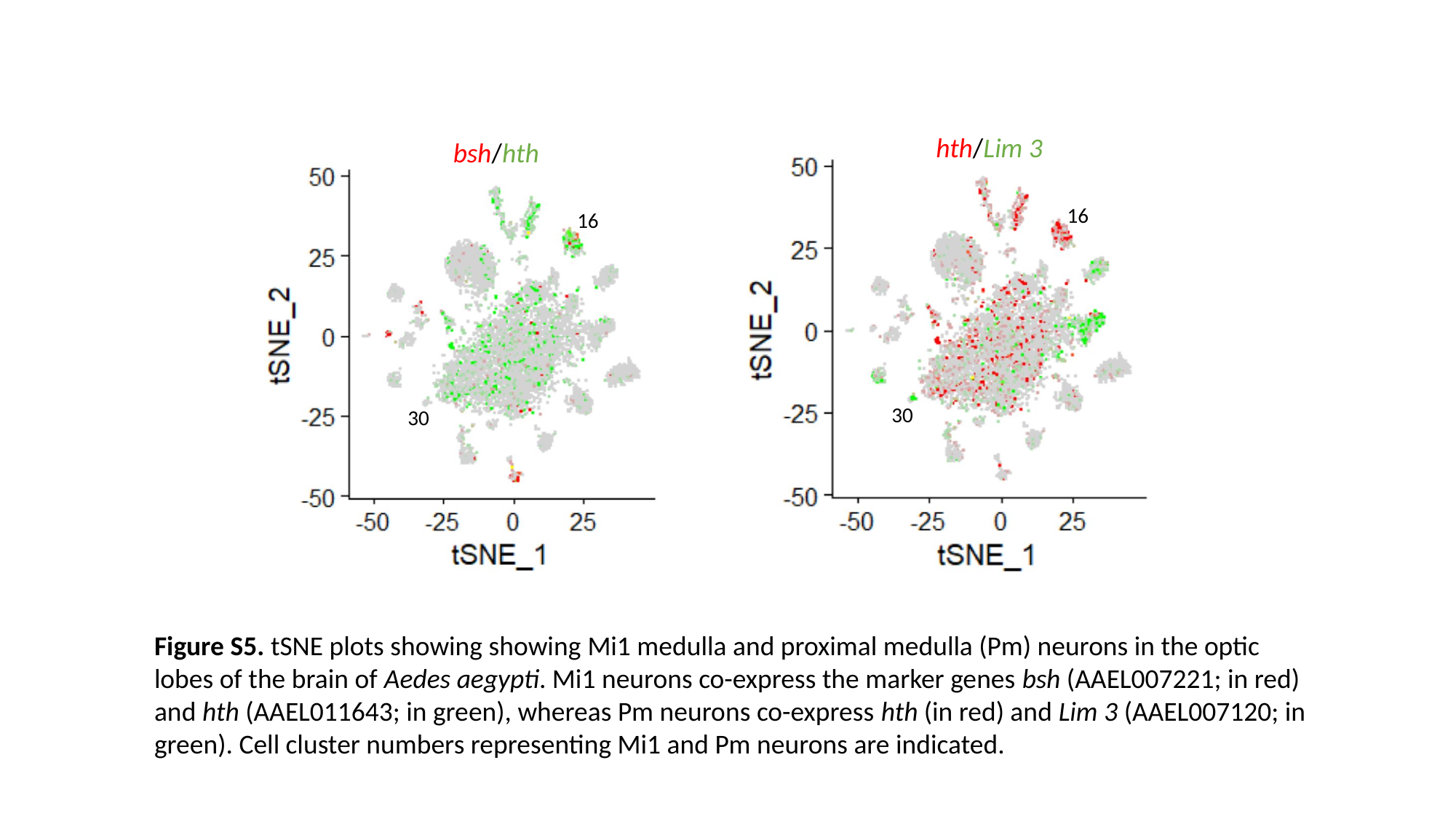

hth/Lim 3
30
bsh/hth
16
16
30
Figure S5. tSNE plots showing showing Mi1 medulla and proximal medulla (Pm) neurons in the optic lobes of the brain of Aedes aegypti. Mi1 neurons co-express the marker genes bsh (AAEL007221; in red) and hth (AAEL011643; in green), whereas Pm neurons co-express hth (in red) and Lim 3 (AAEL007120; in green). Cell cluster numbers representing Mi1 and Pm neurons are indicated.
